# Adherence to malaria rapid diagnostic test result among healthcare workers in Sokoto metropolis, Nigeria

**DOI:** 10.1186/s12936-019-3094-2

**Published:** 2020-01-02

**Authors:** Aliyu Mamman Na’uzo, Dahiru Tukur, Mu’awiyyah Babale Sufiyan, Adebowale Ayo Stephen, IkeOluwapo Ajayi, Eniola Bamgboye, Abdulrazaq Abdullahi Gobir, Chukwuma David Umeokonkwo, Zainab Abdullahi, Olufemi Ajumobi

**Affiliations:** 1 Department of Paediatrics, Federal Medical Centre, Birnin Kebbi, Nigeria; 2 Nigerian Field Epidemiology and Laboratory Training Programme, Abuja, Nigeria; 3 Department of Community Medicine, Ahmadu Bello University, Zaria, Nigeria; 4 Department of Epidemiology and Medical Statistics, Faculty of Public Health, University of Ibadan, Ibadan, Nigeria; 5 Department of Community Medicine, Federal Teaching Hospital, Abakaliki, Nigeria; 6 Department of Community Medicine, Usmanu Danfodiyo University Teaching Hospital, Sokoto, Nigeria; 7 National Malaria Elimination Programme, Federal Ministry of Health Abuja, Abuja, Nigeria; 8 School of Community Health Sciences, University of Nevada, Reno, USA

**Keywords:** Malaria, Guideline adherence, Diagnostic test, Routine, Healthcare workers, Nigeria

## Abstract

**Background:**

Presumptive diagnosis and prescription of anti-malarial medicines to malaria rapid diagnostic test (RDT)-negative patients is a common practice among health care workers (HCWs) in Nigeria. There is paucity of data on HCWs adherence to RDT result in Sokoto metropolis, Nigeria. The study was conducted to determine HCWs adherence to malaria test result and the influencing factors.

**Methods:**

A cross-sectional study was conducted among 262 HCWs selected by multistage sampling technique from primary and secondary health facilities in Sokoto metropolis. Data on demographic characteristics, adherence to RDT result and its influencing factors were collected from the HCWs. Adherence was categorized into good if adherence score is ≥ 4 and poor if otherwise. Chi-squared test was used to test association between adherence to test results and patients’ fever presentation, expectation to be given anti-malarials, prior HCWs’ case management training, among others. Independent predictors of adherence to RDT results were ascertained.

**Results:**

Respondents’ mean age was 33.5 ± 7.9 years, 190 (72.5%) worked in Primary Health Care facilities, 112 (42.8%) were Community Health Workers, 178 (67.9%) had National Diploma Certificate. The median years of practice was 5.0 (IQR: 3–10) years, while 118 (45.0%) had at most 4 years of practice. Overall, 211 (80.5%) had good adherence to RDT results. About 108 (89.3%) of HCWs who had training on malaria case management and 35 (89.7%) certificate holders had good adherence to RDT results. Predictors of adherence to test results were presence of fever in the patient [adjusted odds ratio (aOR): 2.53, 95% confidence interval (CI) 1.18–5.43], patients’ expectation to be given anti-malarial medicines by the HCW (aOR: 3.06, 95% CI 1.42–6.58) and having been trained on malaria case management (aOR: 2.63; 95% CI 1.26–5.44).

**Conclusion:**

High level of adherence to RDT results among HCWs in Sokoto metropolis could be attributed to prior malaria case management training and HCWs’ confidence in the national treatment guidelines. Continual training and supportive supervision of HCWs on malaria case management might optimize the current level of adherence to RDT results in Sokoto metropolis, Nigeria. Similarly, patients/caregivers’ health education could aid better understanding of the need for anti-malarials thus reducing unnecessary demand.

## Background

The World Health Organization (WHO) estimates that about 219 million cases of malaria occurred worldwide [1]. India and 15 countries in sub-Saharan Africa carried almost 80% of the global malaria burden. Nigeria, a sub-Saharan African country accounted for 25% of this burden and 19% of global malaria-related death [1].

In Nigeria, malaria accounted for approximately 60% of outpatient visits and 30% of admissions in 2015 [2]. It contributes about 11% of maternal mortality, 25% of infant mortality and 30% of under-five mortality [2]. It is estimated that about 110 million clinically diagnosed cases of malaria and nearly 300,000 malaria-related childhood deaths occur each year in Nigeria [2].

In 2010, the WHO recommended parasitological confirmation of all suspected malaria cases prior to treatment, this was to mitigate the problem of misdiagnosis and mismanagement of suspected malaria cases [3]. Until the introduction of the rapid diagnostic test (RDT), the diagnosis of malaria has relied mainly on clinical suspicion and microscopic examination of peripheral blood smears where available [4]. Clinical diagnosis of malaria without parasitological confirmation can lead to misdiagnosis of non-malaria febrile illnesses, and eventual selective pressure on currently effective anti-malarial, the artemisinin-based combination therapy (ACT) [5]. In Nigeria, blood smear malaria microscopy, is not readily available or feasible at low-level and peripheral health care facilities because of unavailability of the required skilled manpower, accessories, reagents, its high cost and irregular electricity supply. However, RDT is advantageous in terms of acceptability, availability, ease of use and interpretation of result. The test does not require electricity, and has very high diagnostic accuracy [4–7].

False negative RDT results, persistence of symptoms despite negative RDT result, patients’ pressure and demand for treatment of febrile illness with anti-malarial despite negative RDT result, have been reported to influence healthcare workers’ non-adherence to test result [8, 9]. Studies have shown that the proportion of health care workers prescribing anti-malarial to patients with negative RDT result varies and can be as high as 72% in some settings [10, 11]. Though there is a reported use of RDT for most cases of patients with symptoms of malaria who seek healthcare in health facilities in Sokoto metropolis—Nigeria, but there is paucity of data on health care workers’’ adherence to RDT results in Sokoto Metropolis, Nigeria. This study was conducted to determine HCWs adherence to RDT results and factors influencing their adherence to the test result in this sub-urban community. This will provide a broader understanding of the gravity of prescription of anti-malarial medicines to patients with RDT negative results and informing the development of context specific strategy for optimizing compliance to RDT result in the study area.

## Methods

### Study area

**Fig. 1:**
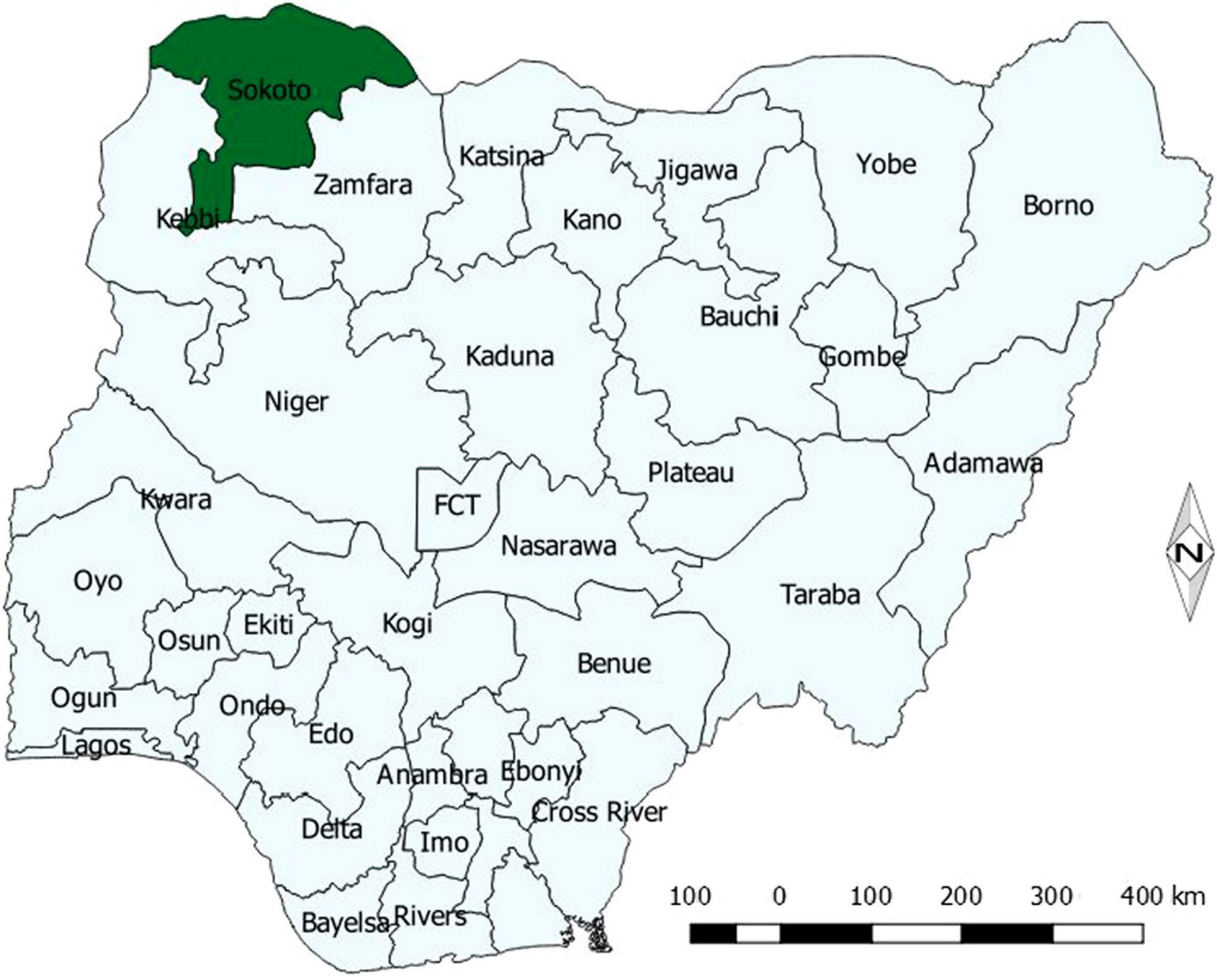
Map of Nigeria highlighting the location of Sokoto State in green within the North western part of Nigeria. Developed using QGIS version 2.4.0 a free GIS software

This study was conducted in Sokoto metropolis, Sokoto State, Nigeria. Sokoto State is located in North-West, Nigeria between longitude 3° and 7° E and latitude 10° and 14° N of the Equator (Fig. 1). According to the 2018 projected population, the metropolis has a total population of 858,005 (based on 2006 population census) distributed in 3 Local Government Areas (namely: Sokoto-North, Sokoto-South and Wamakko). The predominant tribes are Hausa and Fulani while Islam is the main religion. Agriculture, petty trading and craftsmanship are the main occupations of the people [12].

The prevalence of malaria in Nigeria has been found to be 22.6% by RDT [13]. This varies across the six geopolitical zones of the country and across the various states in the zones. In the north-west zone of the country malaria prevalence is 33.8%. In Sokoto State, malaria is hyper-endemic with RDT prevalence of 36.4% and transmission season from January to August [13, 14]. The arrangement of houses within the metropolis is irregular with poor environmental sanitation. This leads to the development of water pools/retention sites during the rainy season, thus providing favourable breeding sites for mosquitoes. Sokoto metropolis has 1379 health care workers of different cadres spread across both public and private health facilities.

The Nigerian health system classifies the public health facilities into primary, secondary and tertiary health facilities. In Sokoto metropolis there are 33 Primary Health Care Centres, 3 secondary health care centres and a tertiary health care centre providing care to over a million people. In the Primary Health Centres (PHCs), malaria is treated by community extension workers, nurses, laboratory scientist and pharmacy technicians while physicians do same in secondary and tertiary heath centres.

Beginning in 2010, over 68 million RDT kits have been deployed for use in Nigeria by the National Malaria Elimination Program (NMEP) to various states in the country [15]. Malaria rapid diagnostic test kits are supplied periodically to the Sokoto State Ministry of Health by NMEP and the United State President’s Malaria Initiative (PMI) through United States Agency for International Development (USAID). From the Central Medical Store, the RDT kits are distributed to health facilities using the Direct Delivery and Information Capture ‘DDIC’ to quantify what each health facility requires. By using the retrospective data in prior month, RDTs were supplied subsequently to each health facility based on surveillance data (average number of fever cases) and logistic data (consumption data available in bi-monthly health facility stock reporting forms). At the time of the study, SD-Bioline malaria RDT Histidine-rich protein (HRP-2) kits were in use. The kits detect the presence of HRP-2 antigen of *Plasmodium falciparum*; which accounts for 94% of malaria infection in Nigeria [2]. As done in other states in Nigeria, training and retraining of health care workers on RDT is implemented periodically by the Sokoto State Ministry of Health with the support of PMI/USAID in both private and public health sectors since the adoption of the test and treat policy in 2010 [16]. State Logistic Management Coordinating Unit, Sokoto State Ministry of Health with guidance from Global Fund-funded Nigeria Supply Chain Integration Project (NSCIP) of National Product Supply Chain Management Programme ‘NPSCMP’ Federal Ministry of Health—Nigeria, implements the supply chain system for RDT kits. An RDT positive result in a febrile patient suspected of malaria, qualifies for the use of ACT as the first-line of therapy as recommended by the WHO.

### Study design and population

This was a cross-sectional study conducted among health care workers in Sokoto Metropolis. The study population comprises Doctors, Nurses, Medical laboratory Scientist, Pharmacy technicians and Community Health Workers (CHWs) in primary and secondary public health facilities within the metropolis. At the PHCs, the CHWs are in three categories each with a specific qualification obtained at the end of their training. The Junior Community Health Extension Worker (JCHEW) are the lowest in the ladder and are awarded a certificate at the end of their training. The next are the Community Health Extension Workers (CHEWs) who obtains a national diploma at the end of their training. The last being the Community Health Officers (CHOs) who are equally awarded higher national diploma at the end of their training. The CHEWs constitute the majority of the healthcare workers in the Primary Health Care Centres. The study was carried out from 1st March to 31st May, 2018.

### Eligibility criteria

The inclusion criterion were HCWs whose job prescription in the health facility included diagnosis and treatment of febrile illnesses. HCWs from private and tertiary health facilities were excluded from the study.

### Sample size and sampling method

A sample size of 276 was calculated using a prevalence of 76.5% [17]. At Zα of 1.96, and a precision of 5%. The sampling frame of all the public health facilities in Sokoto metropolis was obtained from the Sokoto state ministry of health. To ensure representativeness, five- primary and one secondary health facilities were randomly selected from each of the three LGAs in the metropolis. Using a sampling frame (N) of all the health care workers providing care to febrile patients, proportionate to size allocation was applied to determine the proportion of each cadre of HCWs. This was multiplied by the sample size to ascertain the actual number of respondents per cadre(n). The sampling interval was calculated by dividing “N” with “n” (N/n). The first HCW was selected randomly using a table of random numbers and subsequently, every nth HCW was selected until the sample size was completed, see Table 1.

**Table 1:** Sampling of health care workers by probability proportional to size

S/no	Cadre of HCW	No. in Health facility	Calculated proportion	No. allocated to health facility (n)	Sampling interval (N/n)
1.	Junior Community Health Extension Workers	175	175/1100 × 276 = 44	44	4
2.	Community Health Officers	36	36/1100 × 276 = 9	9	4
3.	Community Health Extension Worker	279	276/1100 × 276 = 70	70	4
4.	Nurses	267	267/1100 × 276 = 67	67	4
5.	Medical Laboratory Scientist	183	183/1100 × 276 = 46	46	4
6.	Medical Doctors	132	132/1100 × 276 = 33	33	4
7.	Pharmacy Technicians	28	28/1100 × 276 = 7	7	4
8.	Total	1100		276	4

### Data collection

The interview was conducted at the primary and secondary health care facilities in Sokoto metropolis. Data were collected with a pre-tested, semi-structured interviewer-administered questionnaire using mobile data capturing device (Open Data Kit). The questionnaire collected information on respondents’ demographic variables (age, gender, level of education, marital status), cadre, years of practice, previous training on malaria case management, adherence to RDT results in the treatment of malaria and what influenced the treatment of fever with anti-malarial medicine (an artemisinin-based combination) whenever RDT result is negative. Being a cross-sectional study, participants were interviewed once during the survey (Additional file 1).

### Outcome of interest

The primary outcome was the proportion of healthcare workers that adhered to RDT result when treating patient suspected of malaria in the study facility. Adherence to test result was defined as HCW administering ACT to RDT positive patient and withholding such to RDT negative patients. The primary outcome was assessed using five questions as follows: (1) Is RDT result available at the time of treating the patient [Yes/No]. (2) In the last 6 months, would you say you always complied with the test result when treating your patient with suspected malaria [Yes/No]. (3) In the last 6 months, when prescribing medications to your patients suspected of malaria, do you use RDT result to determine who receives or does not receive ACT [Yes/No]. (4) are you confident of treating patient suspected of malaria using the national malaria treatment guideline [Yes/No] and lastly (5) Do you think treatment of malaria according to the test results will reduce unnecessary use of ACTs [Yes/No].

### Data processing and analysis

Data were summarized using descriptive statistics such as frequency and proportions for categorical variables. Measures of central tendency were computed for continues variables namely: mean and standard deviation for age and median and interquartile range (IQR) for years of practice. Responses to adherence related questions were coded as 1 for a positive response and zero for a negative one. The maximum obtainable score was five. A score of at least 80% of the maximum possible score was considered as good adherence and poor if otherwise. Association between adherence and sociodemographic/HCW related characteristics were examined using Chi-square test. The odds ratio and the 95% confidence intervals of the odds ratio were reported. To identify the predictors of adherence, variables found to be statistically significant at 10% level using bivariate logistic regression were included in the multiple logistic regression model. However, the prediction model was based on 5% level of significance. Four models were used to predict the factors associated with HCW adherence to test results. The factors were categorized into socio-demographic and HCW related factors. Model 1 contained the crudes odds ratio of both group of predictive factors, model 2 contained socio-demographic factors that were significant at 10% in model 1, model 3 included HCW-related factors significant in model 1, and model 4 included all the predictors of adherence. To evaluate for the model fit, Hosmer & Lemeshow goodness of fit tests was assessed.

## Results

Overall 276 questionnaires were administered, out of which 262 (94.6%) had complete responses to all the questions and were included in the analysis. The mean age was 33.5 ± 7.9 years. The median years of practice was 5.0 (IQR: 3–10) years. About half, 121(46.2%) had training on malaria case management in the last 6 months preceding the survey. More than half of the respondents were females 155 (59.2%). Majority of the respondents 190 (72.5%) were from primary health facilities and 107 (40.8%) were within the age group 30–39 years. Majority of them were married 108 (71.0%) and 112 (42.7%) were Community Health Workers. More than two thirds 179 (67.9%) of the HCWs had National Diploma certificate and 118 (45.0%) had practiced for at most 5 years (Table 2).

**Table 2:** Socio-demographic characteristics of health care workers in Sokoto metropolis, Nigeria (N = 262)

Characteristics	Frequency	Percentage
Age (years)		
< 30	98	37.4
30–39	107	40.8
40–49	47	17.9
≥ 50	10	3.8
Sex		
Female	155	59.2
Male	107	40.8
Marital status		
Single	63	24.0
Married	186	71.0
Divorced	6	2.3
Widowed	7	2.7
Professional cadre		
CHW	112	42.7
Nurses	65	24.8
Medical Laboratory Scientist/Technicians	46	17.6
Medical Doctor	32	12.2
Pharmacy Technician	7	2.7
Professional qualification		
Certificate^a^	39	14.9
Diploma	178	67.9
Degree/HND	45	17.2
Years of practice		
< 5	118	45.0
5–9	77	29.4
≥ 10	67	25.6
Facility type		
Primary	190	72.5
Secondary	72	27.5

*CHW* Community Health Extension Workers and Community Health Officers, *HND* Higher National Diploma

^a^Certificate in Junior Community Health Extension Worker

### Adherence to RDT results

About 211 (81%) of the respondents had good adherence. Being a certificate holder (OR: 4.38, 95% CI 1.31–14.61), having had training on malaria case management (OR: 3.07, 95% CI 1.54-6.08), presence of fever in the patient consulted by the HCW (OR: 4.59, 95% CI 2.29–9.21) and patients’ expectation to be given anti-malarial (OR: 4.36, 95% CI 2.12–8.95) were statistically significantly associated with adherence to RDT result (Table 3).

**Table 3:** Factors associated with adherence to test result among health care workers, Sokoto metropolis, Nigeria (N = 262)

Characteristics	Adherence		OR (95% CI)
	Good	Total	
Age (years)			
< 35	128 (81.0)	158	1.08 (0.58–2.01)
≥ 35	83 (79.8)	104	1 (ref.)
Sex			
Male	81 (75.7)	107	0.60 (0.33–1.11)
Female	130 (83.9)	155	1 (ref.)
Facility type			
Primary	156 (82.1)	190	1.42 (0.73–2.74)
Secondary	55 (76.4)	72	1 (ref.)
Professional qualification			
Certificate	35 (89.7)	39	4.38 (1.31–14.61)*
Diploma	146 (82.0)	178	2.28 (1.10–4.73)
Degree/HND	30 (66.7)	45	1 (ref.)
Cadre			
CHW	93 (83.0)	112	1 (ref.)
Doctors/Nurses	79 (81.4)	97	0.90 (0.44–1.83)
Medical lab/Pharm tech	39 (73.6)	53	0.57 (0.26–1.25)
Years of practice			
< 5	95 (80.5)	118	1.0 (0.54–1.84)
≥ 5	116 (80.6)	144	1 (ref.)
Had training on malaria case management			
Yes	108 (89.3)	121	3.07 (1.54–6.08)*
No	103 (73.0)	141	1 (ref.)
Stock out of mRDT influenced prescription			
Yes	169 (80.1)	211	0.86 (0.39–1.91)
No	42 (92.4)	51	1 (ref.)
Presence of fever in the patient influenced prescription			
Yes	185 (85.6)	216	4.59 (2.29–9.21)*
No	26 (56.5)	46	1 (ref.)
Expectation of the patient to be given antimalarial influenced prescription			
Yes	115 (91.3)	126	4.36 (2.12–8.95)*
No	96 (70.6)	136	1 (ref.)
Clinical judgement of the HCW influenced prescription			
Yes	184 (81.4)	226	1.46 (0.64–3.33)
No	27 (75.0)	36	1 (ref.)
Availability of alternative diagnostic tool			
Yes	177 (81.9)	216	1.60 (0.76–3.37)
No	34 (73.9)	46	1 (ref.)

* p ≤ 0.05

### Factors associated with health care workers’ adherence to RDT results

The model 1 of the logistic regression revealed that HCWs who were certificate holders in Junior Community Extension Worker were 4 times (OR: 4.4, 95% CI 1.3–14.6) more likely to have good adherence to RDT results than those with tertiary degree holders (B.Sc. or H.N.D). Similarly, HCWs who had attained diploma were 2 times (OR: 2.3, 95% CI 1.1–4.7) more likely to have good adherence to RDT results than those with tertiary degree holders. The likelihood of good adherence to RDT result was higher among HCWs who had received training on malaria case management (OR: 3.0, 95% CI 1.5–6.1) than their counterparts who were not previously exposed to such training. Similarly, HCWs whose patient presented with fever were 4.5 times more likely to have good adherence (OR: 4.59, 95% CI 2.29–9.21). The predictors of adherence to RDT results (model 4) among health care workers were prior training on malaria case management (aOR: 2.63, 95% CI 1.26–5.44), the presence of fever in the patient being consulted by the HCW (aOR: 2.53, 95% CI 1.18–5.43) and expectation of the patient to be given anti-malarial medicine by the HCW even when RDT result is negative (aOR: 3.06, 95% CI 1.42–6.58), see Table 4.

**Table 4:** Predictors of adherence to Malaria RDT results among health care workers, Sokoto metropolis, Nigeria

Characteristics	Model 1	Model 2	Model 3	Model 4
	COR (95% CI)	aOR (95% CI)	aOR (95% CI)	aOR (95% CI)
Age (years)				
< 35	1.08 (0.58–2.01)			
≥ 35	1 (ref.)			
Sex				
Male	0.60 (0.33–1.11)			
Female	1 (ref.)			
Facility type				
Primary	1.42 (0.73–2.74)			
Secondary	1 (ref.)			
Years of practice				
< 5	0.10 (0.54–1.84)			
≥ 5	1 (ref.)			
Professional qualification				
Certificate	4.38 (1.31–14.61)^†^	4.38 (1.31–14.61)		3.59 (0.10–12.10)
Diploma	2.28 (1.10–4.73)	2.28 (1.10–4.73)*		1.89 (0.83–4.14)
Degree/HND	1 (ref.)	1 (ref.)		1 (ref.)
Cadre				
CHW	1 (ref.)			
Doctors/Nurses	0.90 (0.44–1.83)			
Med. Lab/Pharm tech	0.57 (0.26–1.25)			
Had training on malaria case management				
Yes	3.07 (1.54–6.08)^†^		2.58 (1.26–5.29)*	2.63 (1.26–5.44)*
No	1 (ref.)		1 (ref.)	1 (ref.)
mRDT stock level in the facility influenced prescription				
Yes	0.86 (0.39–1.91)			
No	1 (ref.)			
Presence of fever in the patient influenced prescription				
Yes	4.59 (2.29–9.21)^†^		2.83 (1.33–5.98)*	2.53 (1.18–5.43)*
No	1 (ref.)		1 (ref.)	1 (ref.)
Patients expectations to be given antimalarial influenced prescription				
Yes	4.36 (2.12–8.95)^†^		3.18 (1.48–6.80)*	3.06 (1.42–6.58)*
No	1 (ref.)		1 (ref.)	1 (ref.)
Clinical judgement of the HCW influenced prescription				
Yes	1.46 (0.64–3.33)			
No	1 (ref.)			
Availability of alternative diagnostic tool (microscopy)				
Yes	1.60 (0.76–3.37)			
No	1 (ref.)			

Hosmer–Lemeshow p value = 0.185

*COR* crude odds ratio, *OR* odds ratio, *aOR* adjusted odds ratio, *Ref* reference category = 1

^†^Significant at 10%; * p < 0.05

## Discussion

This study aimed to determine health care workers’ adherence to RDT results and factors influencing their adherence to test result. Overall health care workers have good adherence. In addition, the presence of fever in the patient consulted, the expectation of the patient to be given anti-malarial and HCWs’ prior receipt of training on malaria case management were noted as predictors of adherence to RDT results by HCWs, a finding that illustrate both health care related and personal factors affects adherence to RDT results.

The good adherence to malaria RDT result observed in this study is similar to findings of a previous study conducted in a public setting in the six geopolitical zones of Nigeria [18]. It is also consistent with the findings from a systematic review of fourteen studies in sub-Saharan Africa on health care workers’ adherence to malaria test results [8]. The high adherence to RDT result found in the current study could be attributed to the training of health care workers on RDTs by both governmental and non-governmental organizations in the study area shortly after the introduction of the 3Ts (test, treat and track) policy by the WHO and subsequent adoption of the policy by Nigerian government [19, 20]. Studies have shown that when HCWs practice evidence-based medicine, resources are conserved; selective pressure on ACT is reduced, and ultimately, potential early resistance to anti-malarial medicines can be averted [21].

The lower cadre of health care workers who attained certificate and diploma comprising Community Health Extension Workers adhered to malaria RDT result and this outcome is consistent with what was reported previously [8, 22, 23]. Within the context of primary health care settings in Nigeria, CHEWs play a major role in provision of basic health care services and probably had higher likelihood of adherence because of health care practice based on defined guidelines, periodic training and close supervision. These cadre of health workers have been prioritized in the last decade and beyond for supported training with support from Global Fund grant and projects funded mainly by PMI-Malaria Action Programme for States and United Kingdom Department for International Development—Support to National Malaria Programme. Perhaps, the upper cadre of health care workers such as doctors who are more experienced in management of infectious diseases but might be more confident in their presumptive diagnosis of malaria and relied on past experiences resulting in less likelihood to adhere to test results. This was reflected in a study conducted in Cameroon where HCWs assert that they are not treating laboratory results but rather the patients [20]. The implication of this is that health care workers in the upper cadre are more likely to mistrust RDT result [8].

The presumption that all febrile illnesses in patients are caused by malaria is not true and has led to over-prescription of anti-malarial medicines [24]. The presence of fever in the patient consulted by the HCW was found to be a significant predictor of good adherence of HCWs to RDT result. This in contrast to the findings from a Ugandan study where health worker’s clinical beliefs and limited ability to identify alternative causes of fever influenced their adherence to RDT results. A Zambian study however, found no association between HCWs prescription of anti-malarial medicine for negative RDT results and the presence of fever in the patients [17]. The reliance of HCWs on fever in the patient as synonymous to having malaria might be due to lack of training on malaria case management, poor clinical skill of the HCWs or pervading teaching in medical school decades ago that presence of fever means malaria until proven otherwise. Moreover, the use of parasitological diagnosis to confirm the presence of malaria and the application of an appropriate interventions, has resulted in decreased malaria burden over the years [25].

Expectation of the patient to be given anti-malarial medicines by the HCW despite negative RDT result was another positive predictor of good adherence to national guidelines and test-based administration of treatment in this study. The finding implies demonstration of HCWs’ self-efficacy in adhering to national treatment guidelines and their ability to overcome patients’ persuasion to administer anti-malarial treatment without corresponding evidence based on RDT result. This shows the positive impact of almost a decade-long NMEP/Sokoto State Malaria Elimination Programme/PMI-supported malaria case management trainings and accompanying supportive supervision in addressing the menace of over-prescription and irrational anti-malarial use. Notably, perceived patients’ demand on the HCW for unnecessary treatment with anti-malarial and has the potential for over-prescription of anti-malarial with resultant selective drug pressure. Prior study reported disagreeing with clients’ demand for anti-malarial could result in community members/patients’ loss of confidence in HCWs and this has the potential for non-adherence to RDT results especially in profit-making health facilities [19]. On the other hand, patients’ might persuade HCW to prescribe an anti-malarial despite a negative RDT result; according to 2018 Nigeria Demographic and Health Survey, 75.6% of men and 58.6% of women in Sokoto reported that they will still seek for treatment even if their malaria test shows that their fever was not caused by malaria; simply because they do not trust the test result [13].

The finding in this study that HCWs with prior training on malaria case management were more likely to have good adherence compared to those without this, supports earlier studies that reported the relationship between adherence to test result and training [10, 23]. Training on case management is usually a comprehensive package that includes modules on the use of RDT and adherence to test results. This might probably be the reason why good adherence to test results among those who had received training compared to those who did not was observed in this study. Generally, CHEWs are reported to adhere more to RDT results compared to clinicians and nurses, especially if they were adequately trained and supervised [8]. The study is not without limitation. Reporting bias was a limitation because, the data collected were self-reported. This was however minimized by reassuring the respondents before the interview that confidentiality would be maintained.

## Conclusion

High adherence to RDT result among HCWs in Sokoto metropolis might be attributable to prior training on malaria case management and HCWs self-efficacy in adhering to national treatment guidelines. Continual training of HCWs and supportive supervision on case management of malaria might optimize the current level of adherence to RDT results in Sokoto metropolis. Additionally, health education of patients/caregivers could provide better understanding of the need for anti-malarial treatment and reduce demand for anti-malarial medicines when RDT results are negative.

## Supplementary information

**Additional file 1.** Questionnaire on healthcare workers adherence to malaria RDT results in Sokoto metropolis.

## Data Availability

Data can be made available by the corresponding author based on reasonable request.

## Abbreviations

ABU: Ahmadu Bello University
AFENET: African Field Epidemiology Network
CHWs: Community Health Workers
JCHEWs: Junior Community Extension Worker
CHEWs: Community Health Extension Workers
CHOs: Community Health Officers
CI: confidence interval
DDIC: Direct Delivery Information Capture
HCW: health care worker
HCWs: health care workers
LGA: Local Government Area
RDT: malaria rapid diagnostic test
NFELTP: Nigeria Field Epidemiology and Laboratory Program
NMEP: National Malaria Elimination Program
OR: odds ratio
PHCs: Primary Health Care Centres
PMI: United State President’s Malaria Initiative
SHI: Sustainable Health International
USAID: United States Agency for International Development
WHO: World Health Organization
