# Supplementary material for: Adherence to malaria rapid diagnostic test result among healthcare workers in Sokoto metropolis, Nigeria
Source: Malar J. 2020 Jan 2;19:2. doi: 10.1186/s12936-019-3094-2 (PMC6941286; doi:10.1186/s12936-019-3094-2)
Supplement: Supplementary file 1 — Additional file 1. Questionnaire on healthcare workers adherence to malaria RDT results in Sokoto metropolis. [file 12936_2019_3094_MOESM1_ESM.docx]

|  | **Questionnaire on healthcare workers adherence to malaria RDT results in Sokoto metropolis** | *To be administered to health care workers that treat febrile patients* |
| --- | --- | --- |
|  | Introduction and Consent  Good morning Sir/Ma,  I am Dr Aliyu Mamman Na’uzo conducting a study on healthcare worker’s adherence to Malaria Rapid Diagnostic Test (mRDT) result and factors affecting their adherence. I would like to ask you some questions about RDT, whether or not you use the test result when treating a patient suspected of malaria and factors that influence your prescription of antimalarial medicine. I would appreciate it if you can answer the questions as honestly and freely as you can. Whatever information you choose to share with me will be kept in strict confidence. If at any time you don’t want to answer a question, kindly inform me and I will stop the interview or move to the next question. At the end of the interview, your responses will be analyzed and will be used to make recommendations for further improvement on healthcare worker’s adherence to mRDT results.  Do you agree to the interview?  🞎 Yes  🞎 No | |
|  |  |  |
|  | Date of interview |  |
|  | Type of health facility?  🞎 Primary  🞎 Secondary | |
|  | Initials of officer in charge |  |
|  | Local government Area:  🞎 Sokoto - North  🞎 Sokoto- South  🞎 Wamakko | |
|  | **Personal information of respondents** | |
|  | Age (*as at last birth day*):………………………….. | |
|  | Gender:  🞎 Male  🞎 Female | |
|  | Marital Status  🞎 Single  🞎 Married  🞎 Divorce  🞎 Widow  🞎 others | |
|  | Cadre of respondent: |  |
|  | 🞎JCHEW | 🞎 CHEW |
|  | 🞎 CHO | 🞎 Nurses |
|  | 🞎 Med Lab Scientist/Tech | 🞎 Pharmacist/Pharm Tech |
|  | 🞎 Medical Doctor |  |
|  | Highest professional/technical qualification of respondent:…………….. | |
|  | Number of years working as a healthcare worker:………………………….. | |
|  | **Diagnostic tool for Malaria** | |
|  | What tests do you use to diagnose malaria in our facility?  🞎 No test is done  🞎 RDT  🞎 Microscopy  🞎 Others (*please specify*) | |

|  | **Training on Malaria case management** |
| --- | --- |
|  | Have you been trained on Malaria in the last 24 months? (*if ‘’ yes’’ proceed to Q13 if ‘’No’’ proceed to Q14)*  🞎 Yes  🞎 No |
|  | Which kind of training did you receive?  🞎 Training on Malaria case management and RDT  🞎 Training on RDT alone  🞎 Training on Malaria in pregnancy |
|  | **Adherence to mRDT result** |
|  | Is mRDT result available at the time of treating the patient (*Select most appropriate*?  🞎 Yes  🞎 No |
|  | In the last 6 months, would you say you always complied with the test result when treating your patient with suspected malaria (*Select most appropriate*?    🞎 Yes  🞎 No |
|  | In the last 6 months, when prescribing medications to your patients suspected of malaria do you use mRDT result to determine who receives or does not receive ACT (*Select most appropriate*?    🞎 Yes  🞎 No |
|  | Are you confident of treating patients suspected of malaria using the national malaria treatment guidelines (*Select most appropriate*?    🞎 Yes  🞎 No |

|  | Do you think treatment of malaria according to test results will reduce unnecessary use of ACTs (*Select most appropriate)?*  🞎 Yes  🞎 No |
| --- | --- |
|  | **Factors associated with adherence to test results** |
|  | Does stock out of RDT in your health facility influence your antimalarial prescription?  🞎 Yes  🞎 No |
|  | The presence of fever in the patient can influence your decision to prescribe antimalarial?  🞎 Yes  🞎 No |
|  | Expectation of the patient to be given antimalarial may influence your prescription when the test result is negative?  🞎 Yes  🞎 No |
|  | Clinical judgement plays a major role in your prescription of antimalarial to patients when the RDT result is negative?  🞎 Yes  🞎 No |
|  | My prescription is influenced by availability of alternatives to treatment when RDT result is negative?  🞎 Yes  🞎 No |

**End of questionnaire**

*Thank you for finding time for this interview*
